# Combination and distribution characteristics of syndromes related to traditional Chinese medicine in patients with chronic heart failure: Protocol for a clinical study

**DOI:** 10.1097/MD.0000000000021852

**Published:** 2020-09-04

**Authors:** Hui Wang, Jun Zhang, Chun-fen Shi, Jing Jia, Zhi-min Zhang, Jia-jia Sun, Bing-bing Lu

**Affiliations:** a Department of Traditional Chinese Medicine, the Majiagou Hospital of Kailuan; b Department of Internal Medicine, The Majiagou Hospital of Kailuan; c Department of Endocrinology, Tangshan Hospital of Traditional Chinese Medicine, Hebei; d Kailuan General Hospital, Tangshan.

**Keywords:** chronic heart failure, clinical study, protocol, syndrome elements, traditional chinese medicine

## Abstract

**Introduction:**

Chronic heart failure has become one of the main diseases endangering human health in the 21st century. It is characterized by high morbidity and high mortality. With the continuous in-depth study of Traditional Chinese medicine, the treatment of heart failure by Tradional Chinese medicine has made significant progress, especially in improving the clinical symptoms of patients, controlling the development of the disease, and improving the quality of life of patients.

**Methods/design:**

This will be a retrospective, single-blind clinical observational study. All participants will receive chronic heart failure routine treatment and care. The researcher will fill in the case information collection form and collect multiple clinical diagnosis and treatment information.

**Discussion:**

At present, there is very little research on the elements of chronic heart failure syndrome, and more exploration and excavation in this area are needed. So we designed this program. We aim to explore the distribution characteristics of Traditional Chinese medicine syndrome elements and combinations of chronic heart failure patients, and analyze the relationship between syndrome elements and related influencing factors.

**Trial registration:**

ClinicalTrials.gov,ChiCTR2000034555, Registered on 18 May 2020

## 1 Introduction

Chronic heart failure (CHF) has become one of the main diseases endangering human health in the 21st century. It is characterized by high morbidity and high mortality. The pathogenesis of CHF mainly has 2 aspects. On the one hand, it is primary myocardial damage: long-term glucose and lipid metabolism disorders affect the metabolism of myocardium, vascular endothelium, and other tissues, and promote cardiac microvascular endothelial cell proliferation, basement membrane thickening, reduced oxygen utilization, myocardial fibers, and surrounding blood vessels fibrosis, glycoproteins, collagen fibers, triglycerides, and cholesterol deposition in the heart muscle. These abnormal metabolisms lead to myocardial energy metabolism disorders and decreased energy reserves, resulting in damage to myocardial cells and reduced excitability, leading to left ventricular diastolic heart failure, the most common diabetic cardiomyopathy.^[1,2]^ On the other hand, ischemic myocardial damage: coronary atherosclerotic stenosis, myocardial remodeling after myocardial infarction, myocardial pathological changes, atherosclerotic plaque-induced stenosis and ischemic necrosis, myocardial interstitial fibrosis and necrosis, inflammatory cell infiltration, small significant thickening of the intima of the blood vessel wall, precipitation of a large amount of glycosylated protein, as well as changes in nerve fiber reduction, local spindle and spherical thickening of nerve fibers,^[3]^ resulting in weakened ventricular muscle function, decreased filling, and thickened cardiac muscle and enlarged ventricles, heart failure occurs due to ventricular remodeling. In this type of CHF, coronary heart disease, myocardial ischemia, and (or) myocardial infarction are the most common causes of heart failure.

Western medicine treats CHF mainly with ACEI, diuretics, and cardiotonics. With the continuous in-depth study of Traditional Chinese medicine (TCM), the treatment of heart failure by Chinese medicine has made significant progress, especially in improving the clinical symptoms of patients, controlling the development of the disease, and improving the quality of life of patients. Oral Chinese medicine treatment of CHF can relieve symptoms, improve the quality of life of patients, and prolong the survival time of patients. However, there are also many problems. For example, TCM does not have enough research on the essence of syndromes, which makes the differentiation of heart failure lack norms and standards. Syndrome elements refer to the disease nature and location determined by syndrome differentiation, and are the elements that constitute the name of the syndrome. Syndrome elements are the basic diagnostic unit rather than the classification scheme of diseases. Syndrome elements can have certain combination rules, and there can be overlapping and covering relationships between some syndrome elements.^[4]^ Syndrome elements are the smallest unit of syndromes, and each syndrome element has specific symptoms different from other elements.^[5]^ Syndrome elements can succinctly reflect the location and characteristics of the disease, which is convenient for doctors to flexibly combine the syndrome diagnosis in line with the actual situation of the patient. Therefore, it is very meaningful to apply the advantages of syndrome elements to the exploration of standardized diagnosis and treatment of chronic diseases.^[6]^ At present, most of the research on syndromes focuses on the treatment of CHF. There is a lack of research on the elements of CHF syndrome and the report rate is low. Therefore, we designed this program. We aim to explore the characteristics of the distribution of TCM syndrome elements and combinations of CHF patients, and analyze the relationship between syndrome elements and related influencing factors, and explore the distribution law of syndrome elements, basic pathogenesis, the correlation between syndrome elements and objective indicators of this disease.

## 2 Methods/design

### 2.1 Study population

#### 2.1.1 Source of participants

All cases in this study will come from Majiagou Hospital of Kailuan and Tangshan Hospital of Traditional Chinese medicine. Majiagou Hospital of Kailuan was founded in 1908 and has a history of 104 years. It is a Class-A national public hospital integrating medical treatment, first aid, rehabilitation, prevention and, health care. It is a designated hospital for employees’ medical insurance, urban residents’ medical insurance, Kaiping District New Rural Cooperative Medical Insurance, and provincial and municipal work injury hospitals. The departments of this hospital have internal medicine, surgery, obstetrics and gynecology, emergency department, ENT, stomatology, preventive health care, community health service center, traditional Chinese medicine, rehabilitation, and so on. This hospital has x-ray machines, CT, CR, multiorgan color Doppler ultrasound, Holter monitoring, large-scale biochemical analyzers, and other inspection and inspection equipment. It can meet various clinical needs. Tangshan Hospital of Traditional Chinese medicine was founded in 1972 and is a comprehensive three-level A Chinese medicine hospital. It is a 3-level traditional Chinese medicine hospital integrating medical treatment, teaching, scientific research, prevention, and health care, and a national model Chinese medicine hospital. The hospital has 600 beds and 750 open beds, equipped with a fully digital gastrointestinal machine, computer x-ray radiography DR system, Konica REGIUS computer x-ray radiography CR system, Japan 7600–20 automatic biochemical analyzer, Japan Olympus AU400 biochemical analyzer, French Mérieux bacteria identification instrument, BD automatic blood culture instrument, American TP700 automatic blood coagulation instrument, Japanese blood cell analyzer, Greek automatic five-class blood cell analyzer, urine analysis Medical equipment such as blood gas analyzer, Roche electrochemiluminescence meter, blood rheometer, and so on. It can meet the testing of all the indicators required by the test.

#### 2.1.2 Diagnostic criteria

Regarding the diagnostic criteria for CHF, we will refer to the Framingham diagnostic criteria for heart failure (Table 1).^[7]^ Primary or secondary criteria include: body weight loss ≥4.5 kg after treatment for >5 days. Those who meet 2 main criteria, or meet 1 main criterion and 2 secondary criteria can be diagnosed as heart failure. Our target cases for this study are 500 cases.

**Table 1:**
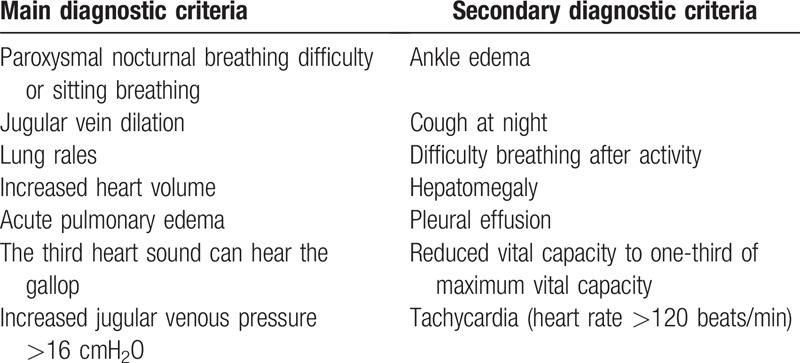
Diagnostic criteria of Framingham heart failure.

### 2.2 Inclusion/exclusion criteria

The inclusion criteria are defined as: meet the diagnostic criteria of Western medicine CHF; cardiac function classification (grading) belongs to patients of grade 2–4 (Table 2)^[7]^; age between 45 and 85 years’ old; those who have voluntarily signed the informed consent. The exclusion criteria are defined as: patients with severe diseases such as malignant hypertension, severe arrhythmia, acute myocardial infarction, and cerebrovascular accident; those who have recently had acute infection, trauma, surgery, diabetic ketoacidosis, and hyperosmolar coma; patients with severe primary diseases such as brain, liver, kidney and hematopoietic system, mental illness, and abnormal cognitive function; patients with tumor and other organ failure.

**Table 2:**
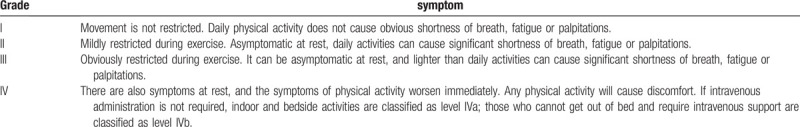
NYHA cardiac function grading standards.

### 2.3 Ethics and informed consent

The research protocol of this clinical trial will be submitted to the ethics committee of Tangshan Hospital of Traditional Chinese medicine for review. The study protocol will be approved by the ethics committee of Tangshan Hospital of Traditional Chinese medicine. We will not start recruiting participants without the approval of the ethics committee. We will develop a detailed informed consent form. We will explain the procedure and precautions of this trial to all participants who are going to participate in this trial. After that, all participants will be required to sign an informed consent form for this study before participating in this trial. Otherwise, it will not be included in this trial.

### 2.4 Study design

This will be a retrospective, single-blind clinical observational study. All participants will receive CHF routine treatment and care. The researcher will fill in the case information collection form and collect multiple clinical diagnosis and treatment information. The details are as follows: basic information: medical record number, time of admission, name, gender, age, and so on; brief medical history: symptoms, signs, tongue, pulse, medical history, CHF course, physical examination, diagnosis, among others; auxiliary examination: (glycosylated hemoglobin, HbAlc), triglyceride (TG), cholesterol (cholestero), N-terminal pronatriuretic peptide, left ventricular ejaculation Blood fraction (left ventricular ejection fraction), liver and kidney function; according to the sum of the contribution of each syndrome to related syndromes, the syndromes are judged. We will establish a database, check every 10 medical records entered, and revise them in time to ensure the accuracy of the data.

### 2.5 Conventional treatment

1. Cause treatment: Control risk factors such as hypertension, diabetes, and use antiplatelet drugs and statins for secondary prevention of coronary heart disease;
2. Improve symptoms: Adjust the usage and dosage of diuretics, nitrates, and cardiotonics according to the condition;
3. Correct use of neuroendocrine inhibitors: Increase from a small dose to the target dose or the maximum dose that the patient can tolerate.
4. Monitoring drug reaction: For patients with reduced water and sodium retention, the diuretic dose can be gradually reduced or small dose maintenance treatment, and it is difficult to completely stop the drug in the early stage. The daily weight change is a reliable indicator for detecting the effect of diuretics and adjusting the dosage, and can detect fluid retention early. In diuretic treatment, sodium intake should be limited (<3 g/day). Patients who use positive inotropic drugs can be changed to digoxin after being discharged from the hospital, and digoxin is stopped for those who have repeated symptoms of heart failure, which can easily lead to aggravation of heart failure. If anorexia, nausea, or vomiting occurs, the digoxin concentration should be measured or the drug should be discontinued tentatively. ACEI (or ARB) increases the dose every 1 to 2 weeks, while monitoring blood pressure, blood creatinine, and blood potassium levels, if blood creatinine is significantly increased (>265.2 μmol/L [3 mg/dL]), hyperkalemia (>5.5 mmol/L) or symptomatic hypotension (systolic blood pressure <90 mmHg) should stop ACEI (or ARB). Patients with stable disease, no fluid retention, and heart rate ≥60 beats/minute can gradually increase the dose of β-blockers. If the heart rate is <55 beats/min or accompanied by symptoms such as dizziness, the dose should be reduced.
5. Monitoring frequency: Patients should self-check their weight, blood pressure, heart rate, and register every day. After discharge from the hospital, follow-up once every 2 weeks to observe symptoms and signs, review blood biochemistry, and adjust the type and dosage of drugs. After the condition is stable for 3 months and the drug reaches the optimal dose, follow-up visits are performed once a month.

### 2.6 Statistical analysis

We plan to use the Chinese version of SPSS 25.0 software to perform statistical analysis on the data collected in the database (SPSS Inc, Chicago, IL). Categorical variable data (ie, count data) are expressed by rate or composition ratio, and compared by *χ*^2^ test, normal distribution measurement data are expressed by mean ± standard deviation (X ± S), and rank data are expressed by rank sum test. In the statistical results, *P* < .05 indicates that the difference is statistically significant, *P* < .01 indicates that the difference is statistically significant, and *P* > .05 indicates that the difference is not statistically significant.

### 2.7 Data safety and management

The trial will be reviewed by the ethics committee of the research institution every 3 months. The committee members of the organization will review the report generated by the researcher to determine whether other measures are needed. This may include corrective actions, interim reviews, violations of stop rules, or the need to communicate out-of-scope data to providers or patients. All reports will be provided to them as the review continues. Any decision to terminate the trial early will be decided by the superior unit of this research institution.

## 3 Discussion

CHF is a myocardial injury caused by any causes such as myocardial infarction, cardiomyopathy, hemodynamic overload, inflammation, and so on, resulting in changes in myocardial structure and function, and finally resulting in hypofunction of ventricular pumping or filling. The main clinical manifestations are dyspnea, fatigue, and fluid retention. CHF refers to a state of persistent heart failure that can be stabilized, worsened, or decompensated. The goal of treating heart failure is not only to improve symptoms and improve the quality of life, but also to address the mechanism of myocardial remodeling, delay and prevent the development of myocardial remodeling, and reduce the hospitalization rate and mortality of heart failure. The adverse reactions of Western medicines related to CHF have restricted their clinical application, and traditional Chinese medicine has its unique advantages in the treatment of CHF, and there are fewer adverse reactions of medicines. Therefore, the treatment of heart failure with integrated Chinese and Western medicine has gradually attracted the attention of the medical community. However, the distribution of TCM syndromes of chronic heart failure is extremely complicated. Traditional Chinese medicine often lacks unified, objective, and evidence-based standards for disease differentiation, diagnosis, and treatment. Therefore, only a few common basic syndrome types can be selected for statistical analysis. This means that this type of research is difficult to cover all syndrome types, which brings difficulties to the study of the distribution law of CHF syndrome types, and also limits the clinical application and promotion of TCM to a certain extent. The syndrome elements in TCM reflect the essential law of disease and pathology, a high-level summary of the location and nature of the disease, and are the smallest unit of TCM diagnosis and treatment. The study of syndrome elements is to extract and decompose complex clinical syndrome differentiation, which is more conducive to grasping the essence of the disease. Some complex syndrome types can be regarded as the corresponding combination of multiple basic syndrome elements. Our research on the combination of basic syndrome elements helps to explore the nature of the complex syndrome types of the disease, perform statistical research on the part of TCM syndrome elements and the relevant indicators of western medicine that are objective, and explore their internal relevance. It is of great significance to guide the clinical diagnosis and treatment of TCM based on the statistical results, and to lay the foundation for the scientific and objectivity of the treatment of heart failure by TCM. At present, there is very little research on the elements of CHF syndrome, and more exploration and excavation in this area are needed. So we designed this program. We aim to explore the distribution characteristics of TCM syndrome elements and combinations of CHF patients, and analyze the relationship between syndrome elements and related influencing factors.

## Author contributions

XXXX.
